# The 'global health' education framework: a conceptual guide for monitoring, evaluation and practice

**DOI:** 10.1186/1744-8603-7-8

**Published:** 2011-04-18

**Authors:** Kayvan Bozorgmehr, Victoria A Saint, Peter Tinnemann

**Affiliations:** 1 Department for International Health Sciences; Institute for Social Medicine, Epidemiology and Health Economics; Charité - University Medical Center, Berlin, Germany

## Abstract

**Background:**

In the past decades, the increasing importance of and rapid changes in the global health arena have provoked discussions on the implications for the education of health professionals. In the case of Germany, it remains yet unclear whether international or global aspects are sufficiently addressed within medical education. Evaluation challenges exist in Germany and elsewhere due to a lack of conceptual guides to develop, evaluate or assess education in this field.

**Objective:**

To propose a framework conceptualising 'global health' education (GHE) in practice, to guide the evaluation and monitoring of educational interventions and reforms through a set of key indicators that characterise GHE.

**Methods:**

Literature review; deduction.

**Results and Conclusion:**

Currently, 'new' health challenges and educational needs as a result of the globalisation process are discussed and linked to the evolving term 'global health'. The lack of a common definition of this term complicates attempts to analyse global health in the field of education. The proposed GHE framework addresses these problems and presents a set of key characteristics of education in this field. The framework builds on the models of 'social determinants of health' and 'globalisation and health' and is oriented towards 'health for all' and 'health equity'. It provides an action-oriented construct for a bottom-up engagement with global health by the health workforce. Ten indicators are deduced for use in monitoring and evaluation.

## Introduction

Today, health is acknowledged as a complex and global issue [1]. The globalisation process has reduced barriers to transworld contacts and enabled people to become 'physically, legally, culturally, and psychologically' engaged with each other in 'one world' [2]. The reduction of barriers has been facilitated by the spread of supraterritorial processes, whose impacts, however, always 'touch down' in territorial localities [2].

Models describing the health impacts of globalisation have been formulated [3]. Strong linkages between globalisation and health have been demonstrated by the Globalisation and Knowledge Network of WHO and evidence-informed policy recommendations for action on the social determinants of health have been formulated [4]. These recommendations are strongly linked to the rebirth of the values and principles of the primary health care approach [5] as the strategy to counter the territorial health impacts of supraterritorial processes.

The outlined change in perceiving health as a global issue is reflected by the evolution of the term 'global health'. While, until recently, health issues beyond national boundaries were primarily addressed in the context of development aid, infectious disease or charity missions [6], a noticeable change has occurred. Today, health issues are perceived more strongly in terms of international interdependency, with concepts ranging from health as an instrument of foreign policy [7] or national security [8] to health as a human right and concern of solidarity [9].

### From perceptions to implications

Beaglehole and his colleagues (2004) outline the implications of the perception of global health on human resources for health [10]. He argues that the health workforce is not in a position to respond effectively to the challenges of our time, mostly because the quantitative and qualitative capacity of the health workforce has not kept pace with changing needs. In qualitative terms he argues that '[..] the global health challenges in this new era require a health workforce with a broad view of public health, with an ability to work collaboratively across disciplines and sectors and with skills to influence policy-making at the local, national, and global level [..]' [10]. If we expect to prepare the future health workforce for these challenges, their training has to address new educational needs.

#### New educational needs?

Knowledge and competencies in the areas of international migration, cross-cultural understanding, emerging and re-emerging infectious diseases, non-communicable diseases, social and transborder determinants of health, health inequities and inequalities, global health organisations and governance, human rights, medical peace work, environmental threats and climate change have become increasingly important in our globalising world - even for those providing care for individuals [11-17].

Universities in the United Kingdom (UK) [13], the Netherlands and Sweden [11,18] as well as Canada [19] and the United States of America [20] have realised the importance of teaching undergraduate medical students about international or global health issues and this teaching has become embedded in medical curricula to different extents. While there are considerable regional differences regarding contents, priorities, concepts and orientations of teaching in this field, a commonality in many of these developments is that they were student driven [13,21,22].

In Germany, generally speaking, it appears that educational institutions have shown little initiative to date in addressing international or global issues, particularly in medical education [23].

International or global perspectives on the aetiology of disease and illness have so far not been explicitly considered, nor mentioned among appeals in recent history [24-26] calling for public health to have a higher priority in German medical education.

Isolated historical appeals have been made by representatives of tropical medicine to prioritise international health in medical education and introduce 'Medicine in Developing Countries' in curricula [27]. Though sustainably successful on a local institutional level, these developments have mainly occured in the rather narrow context of education for foreign medical students from Asia, Africa or Latin America [27] who mostly repatriated after their studies.

It remains yet unclear whether international or global aspects are sufficiently addressed within medical education in Germany under the latest Licensing Regulations [28], especially in respect to the perceived new educational needs outlined above and their different spheres of competence (*knowledge, skills and attitudes*).

Therefore, we have endeavoured to analyse the state of global health in medical education in Germany using the available evidence. As a starting point, we developed a framework for conceptualising 'global health' education (GHE) and to guide monitoring and evaluation of educational interventions and reforms through a set of key indicators which characterise GHE.

## Mapping the conceptual framework of 'global health' education

To map a conceptual framework for GHE requires critical reflections on definitional, translational and practical aspects of global health, both in general and in the field of education. The definitional problems involved in the descriptor global health are discussed in depth elsewhere [29] and it has been shown that the object of global health mainly depends on the question of how the term 'global' is conceptualised. The diversity of what is understood to be 'global' [29] obviously entails evaluation challenges, however, it is crucial that an analytical framework minimises redundancy and provides clarity about the object of the assessment. Such a framework does not exist up to now due to the absence of a commonly used or even agreed definition [29,30].

### The 'global health' education framework

Attempting to overcome the evaluation challenges, we propose in the following a framework based on existing applicable definitions and models. We hereby differentiate "object", "orientation", "outcome" and "methodology" of education in global health.

**Figure 1:**
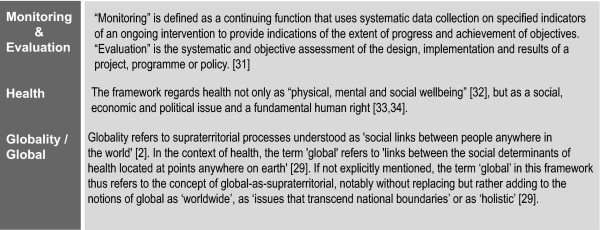
**Definitions**.

For the purpose of the GHE framework, we define the terms monitoring and evaluation [31], health [32-34] and global [29] as illustrated in Figure 1.

#### Adopted key characteristics of existing 'global health' definitions

The framework adopts the key characteristics of the 'global health' definition of Rowson and colleagues (Table 1). This definition includes the developing country heritage of the term 'international health' as well as the new emphasis on the impact of globalisation, i.e. also on industrialised countries. At the same time the authors offer some clarity about the object of global health and the types of knowledge required to practice this field. Their definition broadens global health into the areas of research and education as a cross-disciplinary field, building upon methods from public- and international health sciences. The outcome of an engagement in the field of global health, according to their definition, is the understanding of various social, biological and technological relationships that contribute to health improvements worldwide. *(Rowson M, Hughes R, Smith A, Maini A, Martin S, Miranda JJ, Pollit V, Wake R, Willott C, Yudkin JS: Global Health and medical education - definitions, rationale and practice, 2007, unpublished - quoted in full length in *[29]*, p.3*).

**Table 1:** Key characteristics of 'global health' education

Category	Characteristics *^/^**^/+^	Implication	Rationale
**Object**	Focuses on social, economic, political and cultural forces which influence health across the world*	Learning opportunities in 'global health' focus on the underlying structural determinants of health	To ensure that educational interventions cover the social, economic, political and cultural aetiology of ill health, and not merely its disease-oriented symptoms on a global level
	
	Concerned with the needs of developing countries; with health issues that transcend national boundaries; and with the impact of globalisation *	Learning opportunities in 'global health' link territorial up to supraterritorial dimensions of underlying structural determinants of health	To ensure that educational interventions clarify the links between territorial health situations (either domestic ones and/or situations in other countries) and their underlying transborder and global determinants

**Orientation**	Towards 'health for all' **^/+^	Learning opportunities in 'global health' should adopt and impart the ethical and practical aspects of achieving 'health for all'	To ensure that educational interventions are relevant to people's needs on community, local, national, international and global level
	
	Towards health equity **^/+^	Learning opportunities in 'global health' should emphasise issues of health equity (or health inequity) within and across countries	To ensure that educational interventions orientate on the challenge of achieving health equity worldwide

**Outcome**	Identification of actions	Learning opportunities in 'global health' facilitate the identification of actions (by the student), undertaken to resolve problems either top-down or - more importantly - bottom-up	To ensure that educational interventions foster critical thinking and present options for professional engagement on different dimensions towards 'health for all' and health equity

**Methodology**	Cross-disciplinarity *	Learning opportunities in 'global health' involve educators and/or students from various disciplines and professions	To ensure that educational interventions lead to an understanding of influences on health beyond the bio-medical paradigm and respect the importance of sectors other than the health sector in improving health
	
	Bottom-up learning and problem-orientation	Learning-opportunities in 'global health' require unconventional methods for teaching and learning	To ensure that educational interventions clarify the relevance for the health workforce to deal with transborder and/or global determinants of health

Deduced from: * Rowson et al (2007) cited in [29]; ** Koplan et al (2009) [35]; ^+ ^WHO (1984, 1995, 2005) [38-40]

Denotations of 'global' in this definition are conceptualised as 'worldwide' and as 'transcending national boundaries' (Table 1). With the emphasis on globalisation, however, their definition is also in line with the above proposed concept of global-as-supraterritorial [29], given the term is defined accordingly [2]. The framework accepts the additional priority of achieving health equity and 'health for all' formulated by Koplan and his colleagues [35] or elsewhere as a desirable and crucial but not naturally given [29] condition in GHE.

The adopted key characteristics of the definitions are illustrated in Table 1 and allow to deduce "object", "orientation", "outcome" and "methodology" of an engagement in global health in the field of education.

#### Object

**Figure 2:**
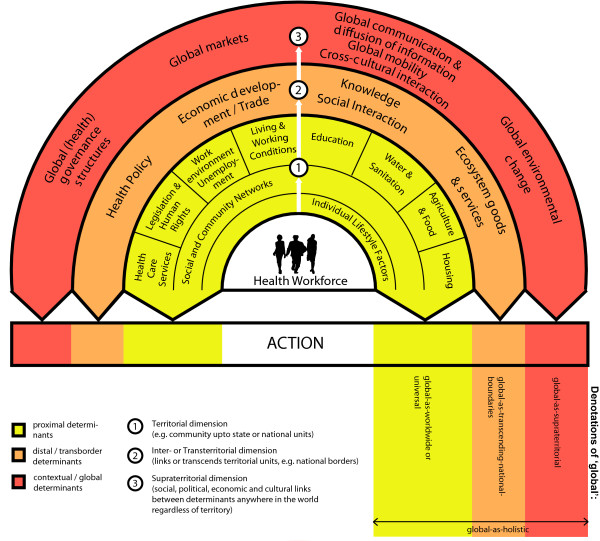
**Framework of 'global health' education**. Adapted from: Dahlgren G & Whitehead M (1991) [36]; Huynen MMTE et al. (2005) [3].

As the object of global health (Table 1) is premised on the engagement with (universal) social, political, economic and cultural forces, our framework builds on the social determinants of health model [36] (Figure 2). Additionally is a 'new' dimension of objects which refer to global as 'transcending national boundaries' and as 'supraterritorial', as captured by the 'globalisation and health model' of Huynen and colleagues [3] (Figure 2).

Both models schematically separate determinants of health in layers, beginning with individual and proximal determinants of health and reaching more distant layers. We refer to the more distant layers of health determinants as transborder (= *inter- or transterritorial*) and global (= *supraterritorial*) determinants.

According to the framework (Figure 2), GHE ideally covers three essential dimensions:

##### 1. Territorial dimension

The territorial dimension predominantly focuses on the universal, proximal social determinants of health (SDH) on community, local, state and national - or in other words - territorial levels. This dimension draws from and overlaps with the public health discipline, which conventionally analyses SDH mainly within a certain territorial unit, such as the domestic nation state (Figure 2).

##### 2. Inter- or Transterritorial dimension

The inter- or transterritorial dimension is focused both on issues that transcend national boundaries *and *on the universal proximal SDH on territorial levels. This dimension draws from the international (public) health discipline. The focus in western medical education is predominantly on surveillance, treatment or containment of infectious (tropical) diseases. In a broader sense, however, the inter- or transterritorial dimension also encompasses the engagement with issues that transcend national boundaries beyond infectious diseases: that is, distal or transborder determinants such as health policies, legal frameworks etc. with inter- or transterritorial nature and/or impact. By accepting the 'historical association with the distinct needs of developing countries' *(Rowson M, Hughes R, Smith A, Maini A, Martin S, Miranda JJ, Pollit V, Wake R, Willott C, Yudkin JS: Global Health and medical education - definitions, rationale and practice, 2007, unpublished)*, this dimension is especially concerned with the delivery and organisation of health care and public health in low- and middle-income countries. In other words, it then includes the territorial dimension of health and development issues in countries other than the domestic country of the student (Figure 2).

##### 3. Supraterritorial dimension

The supraterritorial dimension draws from an engagement with issues related to the *globalisation process *by focusing on global (= *supraterritorial*) influences on health. These are determinants which impact on and thereby link the social determinants of health *anywhere *in the world [29]; but not necessarily *everywhere *or to the same extent [2]. While we analytically distinguish different spheres of social space (Figure 2), we acknowledge that the 'global' is not a domain unto itself, separate from the regional, the national, the provincial, the local, the household [2] and the community.

As such, globality adds to the complexity of social space. It links the SDH and people horizontally *anywhere *in the world and impacts on them through complex pathways [29]. With this understanding of the term 'global', learning about the global dimension *implicitly *includes an engagement with social, political, economic or cultural issues *in *the domestic country of the student, as these issues are linked with SDH *anywhere *in the world by nature and/or impact.

According to our framework (Figure 2), the student is part of the health workforce, which refers to 'all people engaged in actions whose primary intent is to enhance health' [37], without excluding those professions engaged in actions with secondary effects on health (*see Methodology*). This definition includes, but is not limited to, those who promote and preserve health, those who diagnose and treat disease, and health management and support workers, whether regulated or non-regulated [37].

#### Orientation

The framework acknowledges earlier [38,39] and more recent calls by WHO [40] to conceptualise educational programmes for health care providers on the principles of the 'health for all' (HFA) policy. Therefore, the framework proposes that education in global health builds on the three basic values underpinning HFA: (i) health as a fundamental human right; (ii) equity in health and solidarity in action; (iii) participation and accountability [40].

This foundation ensures that educational interventions are socially relevant and orient on people's needs. It is also relevant for GHE because HFA entails: putting health in the middle of development strategies for societies worldwide; linkages between its underpinning principles (i - iii) and the evolution of the term 'global health' and its objects (Table 1); regarding health professional education as a major determinant in realising the HFA objectives [38,39]. Further, primary health care and the social determinants of health can be seen as essential and complementary approaches for reducing inequities in health [41].

According to the proposed framework, GHE should adopt and impart the ethical and practical aspects of achieving 'health for all' with an emphasis on health equity (Table 1).

#### Outcome

The framework does not specify a prescriptive catalogue of topics for global health with detailed educational outcomes, since it is not a curricular proposal. Endless educational outcomes related to the different dimensions could be listed in terms of knowledge, skills and competencies. Generating agreed learning outcomes is urgently needed [42], but remains the responsibility of educator communities within or across countries, with priorities set by schools according to their individual resources and capacities.

For the purpose of monitoring and evaluation, however, the framework suggests to consider the dimensional coverage of educational outcomes in proposals or in curricula as a useful indicator (Table 2).

**Table 2:** Indicators

Category	Indicators	Description	Questions (examples)	Rationale	Methods
**OBJECT**	Dimensional Coverage of Objects	The extent to which the dimensions of the framework are covered by recommendations, curricular proposals or educational interventions.	- Are social determinants of health the predominant object? - Are territorial health issues in the domestic country of the student addressed? - Are territorial health issues in other countries addressed? - Are health issues addressed which transcend national boundaries? - Are supraterritorial health issues addressed?	To analyse the dimensional scope of recommendations/proposals/interventions.	
	
**ORIENTATION**	Health for all		- Are accountability issues of health professionals/the state/civil-society/the private sector/health systems/societies addressed?		
		The extent to which recommendations, curricular proposals or educational interventions explicitly address / explain / cover the underlying principles of ‘health for all’.	- Is the human right to health approach addressed?	To analyse the extent to which the principles of 'health for all' are applied/existent/recommended in teaching and learning.	- (Systematic) Review of curricula/recommendations
			- Is 'health for all' as a concept explained?		- Interviews with deans/chair of faculties
			- Is there a focus on vulnerable groups?		- Questionnaire-based surveys
			- Are equity issues addressed?		
			- Are theoretical and operational principles/mechanisms of solidarity in health/health systems/societies addressed?		
			- Are theoretical and practical principles/mechanisms of participation in health/health systems/societies addressed?		
		
	Equity Focus	The extent to which recommendations, curricular proposals or educational interventions are focussed on health equity.	- Are social theories of equality/inequality addressed? - Are inequalities in health addressed? - Are (avoidable) causes of health inequalities addressed? - Are the operational principles of equity in health/health systems/societies addressed?	To analyse whether recommendations/proposals/interventions have an equity focus.	

**OUTCOME**	Dimensional Coverage of Knowledge	The state or condition of understanding facts (as defined or attained) related to a particular dimension of the framework.	- Is knowledge attained/recommended/proposed related to the object of the field? If yes, in which areas? And on which levels? - ...on territorial levels? - ...on inter -/transterritorial levels? - ...on supraterritoral levels?	To analyse in which areas and dimensions the analysed recommendations/proposals/interventions (aim to) impart knowledge.	- Objective assessments of knowledge/skills/competence among students/graduates
					- Review of curricula/recommendations
	Dimensional Coverage of Skills	The ability (as defined or attained) to use one's knowledge effectively in execution or performance related to a particular dimension of the framework.	- Are skills imparted attained/recommended/proposed related to the object of the field..? If yes, in which areas? And on which levels? - ...on territorial levels? - ...on inter -/transterritorial levels? - ...on supraterritoral levels?	To analyse in which areas and dimensions the analysed recommendations/proposals/interventions (aim to) impart skills.	- Interviews/surveys among deans/chair of faculties
	
	Dimensional Coverage of Competencies	The cluster of knowledge, skills and ability (as defined or attained) to meet complex demands, by drawing on psychosocial resources (including attitudes) in a particular context (related to a particular dimension of the framework).	- Are competencies attained/recommended/proposed related to the object of the field? If yes, in which areas? And on which levels? - ...on territorial levels? - ...on inter -/transterritorial levels? - ...on supraterritoral levels?	To analyse in which areas and dimensions the analysed recommendations/proposals/interventions (aim to) impart competencies.	

**METHODOLOGY**	Multi -/Inter - disciplinarity	The extent to which learning from and with other disciplines is included/addressed/recommended/realised in recommendations, curricular proposals or educational interventions.	- Are educators from different disciplines involved in teaching? - Are students from different disciplines involved in learning? - Is there a diversity in epistemological perspectives on health?	To analyse whether other ('non-medical') schools of thought are prevalent in teaching and learning.	
					- Interviews/surveys among students/graduates/deans/chair of faculties
	Problem-orientation & Bottom-up learning	The extent to which problem-orientation and bottom-up learning is prevalent/applied/realised in recommendations, curricular proposals or educational interventions.	- Are educational strategies based on real problems? - Are educational strategies based on scenarios? - Do educational strategies address the reality of the student? - Do educational strategies link structural determinants of health with the doctor-patient relationship? Or with other levels of professional work?	To analyse the applied/recommended methods in teaching and learning.	- Review of curricula/recommendations

**SOCIOPOLITICAL CONDITIONS & IMPLICATIONS**	Driving Forces	Perceived or evident socio-political conditions, which raise particular implications for health; from the perspective of stakeholders, providers and the target group.	- Are factors mentioned which influence health and health needs? - Which of the dimensions do they cover? - Do these factors have (directly or indirectly) implications for medical education? - Do they raise educational needs? Perceived or evidently?	To analyse which socio-political conditions are regarded as drivers for medical education reform	- Stakeholder analysis (interviews/focus group discussions)
					- Delphi method
	Implications	Perceived or evident implications for medical education which arise from particular driving forces; from the perspective of stakeholders, providers and the target group.	- Which concrete implications are raised by particular driving forces? - Which educational needs are raised? - What is the evidence-base of raised educational needs?	To analyse the implications for medical education among the literature, which arise as a result of particular socio-political conditions.	- (Sytematic) Review of policy documents/recommendations

For the purpose of conceptualising courses, the proposed framework emphasises the identification of actions as a learning objective. That means that acquiring particular knowledge, skills or competencies related to the social aetiology of ill health on different dimensions is ideally followed by the student identifying potential actions to resolve problems on different levels. These actions can be either top-down, i.e. facilitated by actors in higher policy and decision-making fora, but equally - and potentially more important - they can be bottom-up, that is promoted and enforced by the health workforce, for instance by means of addressing the problem via professional, scientific and/or societal action. Resolving problems and identifying actions ideally aims at improvements in health and achieving health equity, in line with the above-outlined orientation of the field.

#### Methodology

Methods put concepts into practice. Therefore, reflecting on adequate methods to link the three elementary dimensions of the framework in practice is crucial. GHE has a cross-disciplinary character, drawing from different schools of thought and perspectives on health (Table 1). Cross-disciplinarity, which we use interchangeably with the terms interprofessionality or multi - or interdisciplinarity, is not constrained to educators alone. It also applies to *the target groups*, ideally comprised of students from different disciplines, professions and academic backgrounds (including political science, economy, law and anthropology etc.). Multi- or interdisciplinary education occurs 'when students from two or more professions learn about, from and with each other to enable effective collaboration and improve health outcomes' [43].

The health workforce is generally trained to work at a circumscribed and limited territorial level, while the medical profession is trained to analyse problems only on the individual level and mainly from the narrow doctor-patient perspective. It is well established, however, that analysing health beyond this narrow perspective is best achieved with bottom-up and problem-oriented approaches [26,44], as illustrated in Figure 2. For the medical profession, this learning approach starts from a problem identified at the doctor-patient or more general territorial level. From here it shifts towards more distal layers for the analysis of the underlying causes of the problem. As outlined above, the aim of the problem analysis is to identify actions to solve a given problem. This promotes critical thinking among the health workforce and is a means to learn and think about the potentials and limits of operationalising the 'health for all' principles in their future professional work.

**Figure 3:**
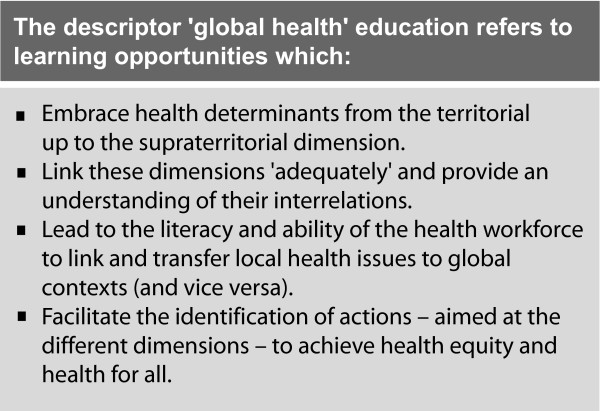
**Summary of 'global health' education**.

**Figure 4:**
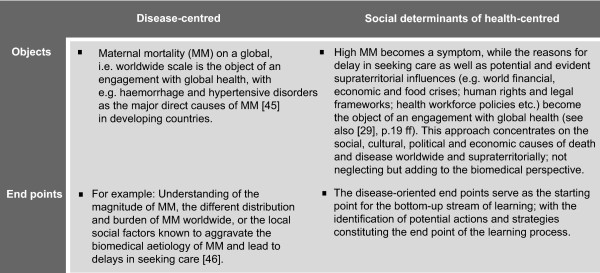
**Key differences between disease-centred and social determinants of health-centred approaches to 'global health' education: The example of maternal mortality**.

The panels summarise the essentials of the above proposed concept of GHE (Figure 3) and illustrate the impact on the object and end points of the learning process compared to conventional approaches to global health, using the example of maternal mortality (Figure 4) [45,46].

#### Perspectives of relevant actors

**Figure 5:**
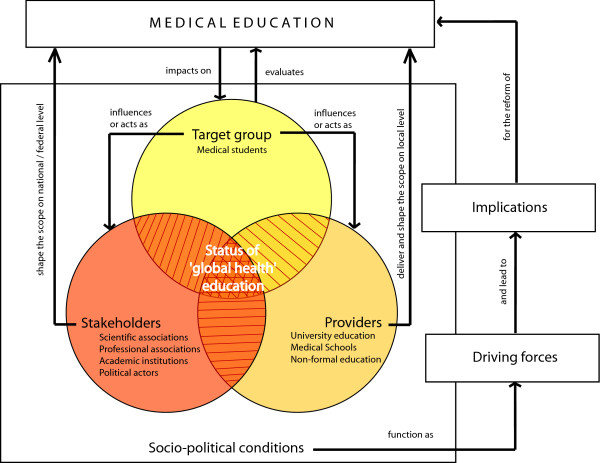
**Perspectives of actors in society with relevance for health professional education: The example of medical education**.

The history of medical education in Germany demonstrates that socio-medical issues in medical training reflect specific socio-political conditions. Changing socio-political conditions function as drivers for reforms of health professionals' education, for example by requiring inclusion of new educational objects (Figure 5).

The nature, importance or consequence of the same socio-political condition might be perceived differently by different actors in society, such as academic associations, deans or medical students. Therefore, the framework suggests that in order to assess the status of eduational interventions, the perspective of relevant actors on both the particular subject of interest and on overall driving forces for education reforms be considered (Figure 5).

#### Indicators

Finally, we deduce ten core indicators from the above framework for the purpose of monitoring and evaluation via different methodological approaches. In Table 2, we define the indicators and provide a set of guiding questions to help decision-making during the assessment of recommendations, curriculum proposals, syllabi or educational interventions.

## Discussion

This framework proposes key characteristics and indicators to facilitate the conceptualisation, evaluation and monitoring of 'global health' education (GHE). It differentiates between "object", "orientation", "outcome" and "methodology" of education in global health. Furthermore, it suggests that a comprehensive approach needs to cover three dimensions of health determinants: build on the 'health for all' principles; focus on health equity; and facilitate the identification of actions to solve health problems in a bottom-up approach within multidisciplinary learning environments.

The GHE framework is not intended to be prescriptive and can be adapted flexibly to local resources or contexts if used to conceptualise courses in practice. It includes examples of indicators to guide the evaluation of educational interventions or the monitoring of curriculum development during education reforms. It further suggests comprehensive consideration of the driving forces for education reform and the different perspectives of relevant actors.

### Points of Controversy

#### Object

Global health is often discussed in the context of the worldwide distribution, prevalence and burden of diseases. The proposed framework does not explicitly take into account major disease-specific aspects of global health nor the leading (direct) causes of worldwide deaths. It does not focus on *global-as-worldwide *health risks [47], but on *global-as-supraterritorial *health risks, i.e. on the social links between the underlying determinants of health risks across the world [29]. As such, education in global health frames particular disease specific aspects and their different distribution, prevalence or incidence patterns as symptoms of social determinants with their according supraterritorial links (Figure 4) .

As such, the framework ensures that GHE of health professionals does not become medicalised by dealing only with curative medicine and health care in countries other than the student's country; an approach more accurately labeled 'global medicine' or 'global health care'.

Similar approaches, which build on a social paradigm, have been described earlier in the field of education (e.g. related to tuberculosis control [48]), shifting the focus from the individual to the community, from physical to social determinants of health, from dependence creating to empowering, from drugs to social interventions and from molecular biology to socio-epidemiology [48]. These would be highly relevant and timely, if applied conceptually and practically to contemporary education in the field of global health.

#### Orientation

It could be argued that in educational interventions a neutral approach is always necessary. However, being neutral is in itself a political decision and not necessarily equivalent to being apolitical. If, firstly, health is accepted as previously defined and, secondly, it is acknowledged that globalisation is not apolitical [2], an apolitical approach towards education in global health becomes literally a paradoxical undertaking (see also [29]).

The different social spheres outlined in the dimensions of the GHE framework (Figure 2) always involve politics, by necessitating processes of acquiring, distributing and exercising social power and entailing contests between different interests and competing values [2] among different actors in society; worldwide *and *supraterritorially.

The political dimension of public health issues - regardless of their dimension - has also been described as a crucial factor for the persistence of know-do-gaps, yet is often neglected by the public health community [49]. The increasing importance given to intersectoral action, for example, acknowledges that achieving health equity requires finding, negotiating and creating opportunities for action and entry points within the health sector and outside of it in the whole of society [41].

From an educational perspective, we believe it is important that students gain political acumen by analysing and determining whose health suffers and 'whose power rises under prevailing practices of globalisation' [2] in order to consider whether alternative policies - aimed at different dimensions - could have better implications for people's health worldwide.

Once this political approach is accepted, GHE could be a means to bring the politics of health back into health professionals education and training. This would, in turn, help to create a health workforce capable of delivering health back into politics; thereby helping to foster, support and facilitate policies towards 'health for all'.

As the orientation of the GHE framework places emphasis on achieving health equity within and across countries, learning opportunities in global health should explicitly deal with health inequities, understood as avoidable inequalities in health [50].

Such health inequities 'mostly point to policy failure, reflecting inequities in daily living conditions and in access to power, resources, and participation in society' [51]. If the focus of education in global health is shifted towards the interface between these inequities and health professionals' role, educational programs might impart a better understanding of 'the power vested in our roles as health professionals and how this power can be used' [52].

Important to note is that the politicisation of education is not equivalent with ideologisation. The approach proposed by the GHE framework does not aim to impose ideologies, thinking patterns and blueprints on the student, but rather, regards politicisation as essential prerequisite for autonomy and impartiality [29].

Learning environments which adopt this framework create space for a student-centred, self-determined, interactive, critical and controversial engagement with global health and the related politics, based on experience and evidence gathered in this field in the last decades worldwide. During this learning process, the students decide autonomously whether 'health for all' and health equity is a utopia or rather an existing heterotopia, which needs their concerted, passionate, long-term and professional engagement to become a mainstream reality worldwide.

#### Outcome

Educational outcomes in the different spheres of knowledge, skills and competence are always a result of complex interactions between numerous factors and thus not always amenable to planning. Therefore, the framework prescribes neither specific learning objectives to be followed in practice nor any topic catalogues to be used as indicators for monitoring and evaluation. For monitoring and evaluation endeavours, it rather suggests to use the dimensional coverage of educational outcomes as an indicator to analyse the extent of globality of existing curricula or recommendations.

By conceptualising an action-oriented framework for GHE in practice, we further aim to initiate debate on more fundamental questions in the context of educational outcomes: Should education in global health inevitably lead to professional specialties or sub-specialties in the field of (public) health sciences? Should education in global health produce a specialised workforce to meet the increasing demand for global health specialists in the labour market or transnational organisations? Should GHE produce global health experts separate from normal health experts?

In the proposed framework, the outcome of education in global health is none of the above. Nor does the framework aim to produce via different career paths a 'globalist health workforce' separate from the 'localist health workforce'. Rather, the framework proposes as an outcome of GHE a health professional, trained in a specific field (e.g. medicine), who understands how their professional work on local levels can feed into or be linked with broader actions in order to impact positively on the SDH on different dimensions. Essentially, the focus of the proposed framework is 'global health' *literacy*, i.e. a fundamental ability of the health workforce to link and transfer local issues to global contexts and vice versa (Figure 3).

The outcome is well described by the term 'activist professional' *(Narayan R: pers. comm.)*, who researches, teaches, works or advocates towards 'health for all' by using their generic professional skills and competencies. Education in global health thus becomes a means to 'mobilise the commitment of the workforce' [5] rather than an end in itself, acknowledging that without this mobilisation the health workforce can be 'an enormous source of resistance to change, anchored to past models that are convenient, reassuring, profitable and intellectually comfortable' [5].

#### Methodology

We admit that, in attempts to link the three dimensions, the complexity of the causal chain increases when analysing determinants of health in more distant layers. The increasing complexity complicates serious attempts to attribute global, i.e. supraterritorial, processes to health risks, morbidity and mortality. In some cases this attempt might not be possible and only hypothetical in nature; in contrast to the analysis of global health risks using the concept of 'global' as worldwide or universal [47]. Nevertheless, it is important to educate students about well-established links and explore unanalysed plausible links, in order to facilitate identification of potential actions via a student-centred approach. GHE as proposed by this framework, thereby goes beyond pure reproduction of facts or problem analysis: it creates space to clarify, discuss or develop opportunities for the health workforce to use their current or future professional activities to influence the determinants of health on different dimensions.

Creating this space could be achieved e.g. by drawing from existing examples of bottom-up activities [5,53], which have successfully influenced policy-making at local, national and international level. Based on these examples, the health workforce explores possibilities to function as professional researchers, educators, practitioners or advocates in health beyond the bio-medical paradigm.

## Conclusions and Implications

The framework presented in this paper provides clarity about key characteristics of education in global health and proposes indicators to guide monitoring and evaluation in the scope of medical education. In a subsequent article [*unpublished*], we use this idealised conceptual framework as an analytical tool to assess publications, educational programs and syllabi in the context of medical education in Germany. We analyse whether, and to which extent, the key characteristics of the framework are represented in public health and international health teaching in German medical education. In doing so, we will assess the state of global health in German medical education and evaluate the applicability of the framework as an analytical tool.

## Competing interests

Financial competing interests

The authors declare that they have no financial competing interests.

Non-financial competing interests

This article has been produced as part of the research thesis of KB at the Institute for Social Medicine, Epidemiology and Health Economics, Charité - University Medical Center Berlin, Germany to earn an academic degree (*Dr.med*).

## Authors' contributions

All authors have made substantial contributions to the manuscript. KB developed the arguments, conceptualised the framework and drafted and revised the manuscript. PT provided critical advice during all steps of the process and revised the manuscript for important intellectual content. VAS reviewed and revised the article for important content related to social determinants of health. All authors have read and approved the final manuscript.

## Authors' information

**KB **(Doctoral candidate) studied medicine in Frankfurt (Germany) and Bangalore (India), undergoing a research fellowship at the Dept. for International Health, Institute for Social Medicine, Epidemiology and Health Economics at the Charité - University Medical Center in Berlin, Germany.

**VAS **(MMSc, BSSc/BHS) is a research consultant, with experience working with WHO in Geneva, universities in Australia and Sweden and with NGOs and research organisations in India.

**PT **(MD, MPH) is the coordinator of the Dept. for International Health at the Institute for Social Medicine, Epidemiology and Health Economics; Charité - University Medical Center, Berlin.

**KB **and **PT **have extensive experience in designing and conceptualising formal and non-formal learning opportunities in global health for medical and non-medical students.
